# Analysis of Prostate Deformation during a Course of Radiation Therapy for Prostate Cancer

**DOI:** 10.1371/journal.pone.0131822

**Published:** 2015-06-29

**Authors:** Takuya Nakazawa, Kunihiko Tateoka, Yuichi Saito, Tadanori Abe, Masaki Yano, Yuji Yaegashi, Hirokazu Narimatsu, Kazunori Fujimoto, Akihiro Nakata, Kensei Nakata, Masanori Someya, Masakazu Hori, Masato Hareyama, Koichi Sakata

**Affiliations:** 1 Department of Radiation Oncology and Medical Physics, Graduate School of Medicine, Sapporo Medical University, Sapporo, Hokkaido, Japan; 2 Department of Radiology, Kushiro City General Hospital, Kushiro, Hokkaido, Japan; 3 Preparatory Office for Proton Therapy Center, Radiation Therapy Research Institute, Social Medical Corporation Teishinkai, Sapporo, Hokkaido, Japan; Innsbruck Medical University, AUSTRIA

## Abstract

**Purpose:**

Accurate analysis of the correlation between deformation of the prostate and displacement of its center of gravity (CoG) is important for efficient radiation therapy for prostate cancer. In this study, we addressed this problem by introducing a new analysis approach.

**Method:**

A planning computed tomography (CT) scan and 7 repeat cone-beam CT scans during the course of treatment were obtained for 19 prostate cancer patients who underwent three-dimensional conformal radiation therapy. A single observer contoured the prostate gland only. To evaluate the local deformation of the prostate, it was divided into 12 manually defined segments. Prostate deformation was calculated using in-house developed software. The correlation between the displacement of the CoG and the local deformation of the prostate was evaluated using multiple regression analysis.

**Results:**

The mean value and standard deviation (SD) of the prostate deformation were 0.6 mm and 1.7 mm, respectively. For the majority of the patients, the local SD of the deformation was slightly lager in the superior and inferior segments. Multiple regression analysis *revealed that the* anterior-posterior displacement of the CoG of the prostate had a highly significant correlation with the deformations in the middle-anterior (*p* < 0.01) and middle-posterior (*p* < 0.01) segments of the prostate surface (*R^2^* = 0.84). However, there was no significant correlation between the displacement of the CoG and the deformation of the prostate surface in other segments.

**Conclusion:**

Anterior-posterior displacement of the CoG of the prostate is highly correlated with deformation in its middle-anterior and posterior segments. In the radiation therapy for prostate cancer, it is necessary to optimize the internal margin for every position of the prostate measured using image-guided radiation therapy.

## Introduction

The goal of radiation therapy is to concentrate radiation doses on the tumor while minimizing exposure of surrounding healthy tissue [1]. This goal can be achieved by using two radiation therapy techniques: three-dimensional conformal radiation therapy (3DCRT) and intensity-modulated radiation therapy (IMRT) [2]. In addition, it is possible to reduce the set-up uncertainty by using image-guided radiation therapy (IGRT), which utilizes a kilovoltage cone-beam CT (CBCT) system mounted on a linear accelerator [3, 4], in combination with the above two techniques.

Planning target volume (PTV) margin, which takes into account both the internal margin (IM) and the set up margin (SM), is utilized during the radiation therapy planning to deliver a prescribed absorbed dose to the clinical target volume (CTV). Minimization of the PTV margin can reduce the risk of toxicity to surrounding normal tissue [5]. PTV margin reduction that does not account for physiological uncertainties (e.g. rectum filling) can lead to biochemical failure of radiation therapy for prostate cancer [6]. The IM is typically calculated based on displacement of the center of gravity (CoG) of the prostate or implant markers in the prostate gland [7, 8]. In radiation therapy for prostate cancer, however, IM does not sufficiently take into account the deformation of the prostate. Accordingly, significant prostate deformations have been demonstrated that can cause differences between the delivered dose and the planned dose [9].

In the published reports, prostate deformation analysis was carried out using computed tomography (CT) or magnetic resonance imaging (MRI) images that were repeatedly acquired during the course of radiation therapy for prostate cancer. Thus, Deurloo *et al*. quantified the deformation of the prostate and seminal vesicles using repeat CT scans and reported that this deformation was small compared to the prostate motion [9]. van der Wielen *et al*. obtained similar results based on deformable image registration (DIR) of CT images [10]. In contrast, Nichol *et al*. detected physiological deformations of prostate greater than 3 mm based on quantification of MRI images [11].

Deuloo *et al*. assumed that prostate deformation occurs only in the direction perpendicular to the surface of the prostate gland [9]. In their study, the deformation was obtained based on chamfer matching of the entire prostate and seminal vesicles. Accordingly, the prostate deformation might have been influenced by the deformation of the seminal vesicles [10]. On the other hand, the accuracy of the DIR approach, which was used to analyze the prostate deformation by van der Wielen *et al*. and Nichol *et al*., varies depending on the algorithm employed [12], and the corresponding discrepancies may have affected the results of the analysis. Moreover, the main aim of the prostate deformation analysis so far was to calculate the standard deviation (SD) of the deformation of the prostate, whereas the correlation between the displacement of the prostate and its deformation has not been previously investigated. Since the IM defined by International Commission on Radiation Units (ICRU) report 62 takes into account the geometric uncertainty in the displacement and deformation of the target, it is important to investigate this correlation.

In this study, we quantified the deformation of the prostate using a simple method that defines the deformation direction and analyzed the correlation between the deformation and displacement of the CoG of the prostate in radiation therapy.

## Materials and Methods

### Patients

Nineteen prostate cancer patients treated between April 2011 and August 2012 with 3DCRT were retrospectively studied. The retrospective analysis was conducted in accordance with the Helsinki declaration, and the study protocol was approved by the medical ethics committee of Kushiro City General Hospital. Patient information was anonymized and de-identified prior to analysis. The tumor clinical stages were as follows: T1, 7 patients; T2, 8 patients; and T3, 4 patients. Eleven patients underwent hormonal therapy before 3DCRT (median duration: 6 months, range: 3–21). All patients were prescribed a dose of 70 Gy in 35 fractions over 50–55 days. They were instructed to empty the rectum and drink 250 ml of water 30 min before the planning CT (LightSpeed RT, GE Healthcare Ltd, Little Chalfont, Buckinghamshire, HP7 9NA, United Kingdom) and treatment delivery for bladder filling. The CBCT images were acquired once every five fractions for a total of 7 datasets per patient. The planning CT and CBCT images were reconstructed using a 2.5-mm slice thickness and a 2.5-mm increment. A single observer contoured the prostate on all CBCT images.

### Prostate volume analysis

Sanguineti *et al*. reported that prostate volume decreases in time under the influence of hormone therapy [13]. To investigate whether prostate deformation includes such a volume change, the prostate volume change during the course of radiation therapy was investigated. The mean and SD of the prostate volume were calculated with a radiation treatment planning system (Eclipse v 6.0, Varian Medical Systems, Palo Alto, CA). The time trend of the prostate volume change was evaluated using the Spearman’s rank correlation coefficient.

### Prostate deformation analysis

The deformation of the prostate was defined as the distance between the prostate surface in the planning CT image and the CBCT image for each fraction. Prostate deformation analysis was performed using in-house developed software.

**Fig 1:**
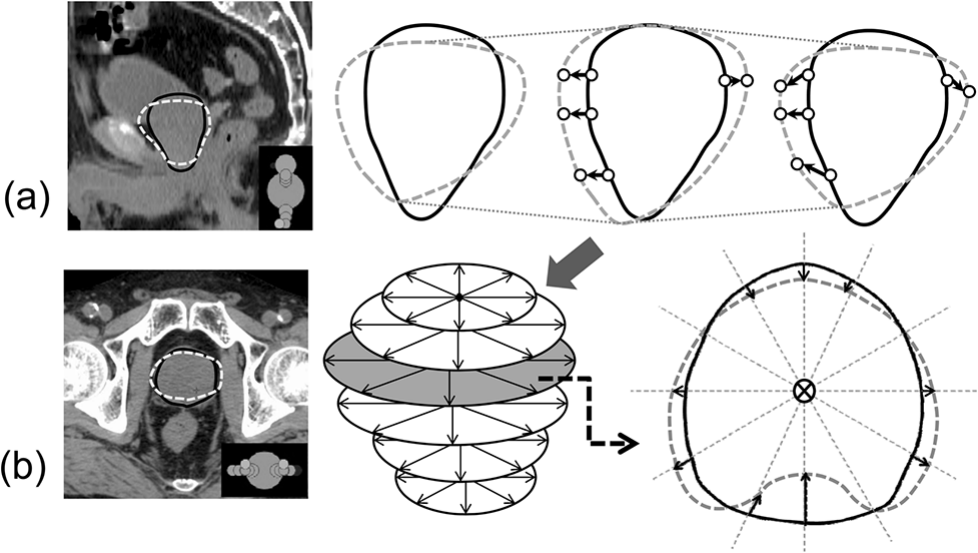
Definition of the proposed prostate deformation quantification metric. The solid and dashed lines represent the prostate contours in the planning CT and CBCT scans, respectively. (a) A 2D representation of the sagittal contours of the prostate. To align the positions of the base and apex of prostate contours in the CBCT scans to those in the planning CT scan, craniocaudal views of the prostate contour in the CBCT scans were either expanded or contracted. They were returned to their original states after the alignment. (b) A 2D representation of the axial contours of a prostate. The cross mark is the CoG of the prostate in a representative slice. The arrows indicate the prostate deformation directions.

**Fig 2:**
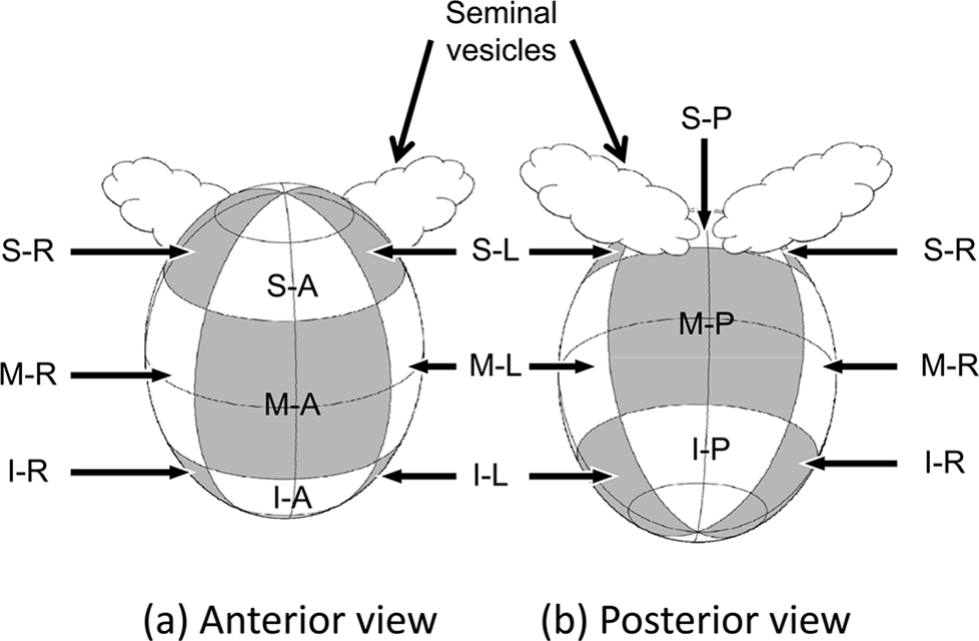
Division of the prostate gland into 12 segments. Superior-anterior (S-A), superior-posterior (S-P), superior-right (S-R), superior-left (S-L), middle-anterior (M-A), middle-posterior (M-P), middle-right (M-R), middle-left (M-L), inferior-anterior (I-A), inferior-posterior (I-P), inferior-right (I-R), and inferior-left (I-L). The prostate was divided into three approximately equal segments in the slice dimension (the first 1/3 of the prostate volume was termed the superior segment, and the last 1/3 was termed the inferior segment) and into four equal segments in each slice (anterior, posterior, right, and left segments).

First, prematching was performed. Rigid bone registration between the planning CT and the CBCT images with contoured prostate was obtained with Velocity AI (Velocity Medical Systems, Atlanta, GA 9), which aligned the two coordinate systems. Next, the contouring data in the planning CT and the CBCT images were transferred to in-house developed software. Prostate displacement was measured as the displacement between the CoG of the prostate in the planning CT scan and the CBCT scans, and the planning CT scan and all the CBCT scans were matched based on the CoG of the prostate translation. To align the slices of the base and apex of the prostate contours in the CBCT scans and the planning CT scan, the craniocaudal views of the prostate contour in the CBCT scans were either expanded or contracted temporarily (Fig 1A). If the number of slices of prostate contours in a CBCT scan was different from that in the planning CT scan, linear interpolation between the slices in the CBCT scan was utilized to match the two numbers. The points at which the prostate contours in the planning CT scan and the CBCT scans intersected with a straight line drawn every 10 degrees through the CoG of the prostate contour for each slice of the planning CT image were calculated as the reference points (Fig 1B). Finally, the craniocaudal views of the prostate contour in the CBCT scans were returned to their original state, and the distances between the corresponding points were measured as local displacements of the prostate (Fig 1B). To evaluate local deformations, the prostate was divided into 12 manually defined segments: superior-anterior (S-A), superior-posterior (S-P), superior-right (S-R), superior-left (S-L), middle-anterior (M-A), middle-posterior (M-P), middle-right (M-R), middle-left (M-L), inferior-anterior (I-A), inferior-posterior (I-P), inferior-right (I-R), and inferior-left (I-L) (Fig 2). The correlation between the displacement of the CoG and the local deformation of the prostate was evaluated using multiple regression analysis (the stepwise method).

### Intra-observer error

To exclude inter-observer errors, a single observer contoured the prostate on all images. To assess the intra-observer error using the method introduced by Deurloo *et al*. [9], prostate images acquired with planning CT and CBCT were recontoured by the same observer. During this process, the observer was blinded to the initial contours. Intra-observer variation results in overestimation of SDs of the local displacement of the prostate. The measured SD of the local displacement (*SD*_*measured*_) can be written as:

$$SD_{measured}^{2}=SD_{intra}^{2}+SD_{actual}^{2}$$ 1

where *SD*_*intra*_ is the intra-observer variation and *SD*_*actual*_ is the actual SD of the local displacement of the prostate. The local displacements between the initial and second contours (*LD*_*i-s*_) and their SDs were calculated using the approach described in the previous section. Given that the error associated with intra-observer variation was introduced twice during this process, these SD values were divided by $\sqrt{2}$:

$$SD_{intra}=\frac{\sqrt{\frac{\sum_{j=1}^{n}{\left\{{\left(LD_{i-s}\right)}_{j}-\bar{LD_{i-s}}\right\}}^{2}}{n-1}}}{\sqrt{2}}$$ 2

where *n* is the number of image sets for each patient. The values of *SD*_*actual*_ were calculated with Eq 1.

## Results

### Prostate volume analysis

**Fig 3:**
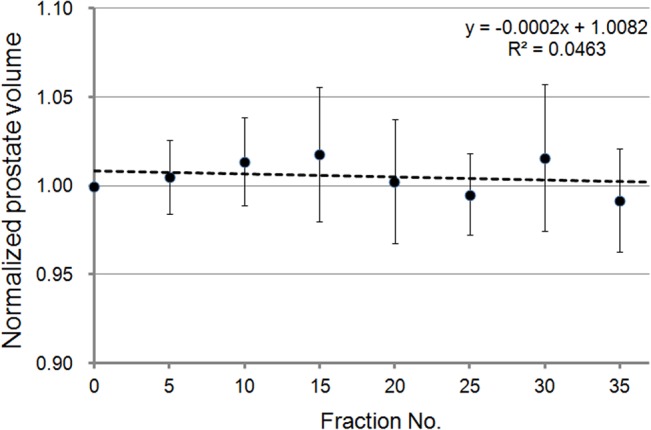
The relationship between radiation therapy fraction number and normalized prostate volume in 19 patients. There was no significant time trend.

The mean values and SDs of the prostate volume were 30.7 ml and 15.9 ml, respectively. The volume normalized with respect to the volume obtained from the results of the planning CT is shown in Fig 3 as a function of the fraction. The normalized volumes were averaged over the 19 patients. The obtained coefficient of determination was 0.0463, and no significant linear time trend was observed (*p* = 0.28). Since the prostate volume did not change during the course of radiation therapy, volume variations did not interfere with the evaluation of the prostate deformation.

### Prostate deformation analysis

Table 1 summarizes the average prostate deformations in the manually defined segments. The maximum average prostate deformation was 1.3 mm in the I-L segment, and the average deformation value was below 1.0 mm in almost all the segments. The maximum absolute prostate deformation was 13.1 mm in the anteroposterior (A-P) direction in the S-P segment of the prostate.

**Table 1:** Values of the average prostate deformation in the manually defined segments.

	Average deformation (mm)			
Prostate segment	Right	Left	Anterior	Posterior
Superior	0.6	1.0	0.4	0.7
Middle	0.2	0.4	0.0	0.3
Inferior	1.1	1.3	0.5	0.8

The SDs of the prostate deformation in the manually defined segments are summarized in Table 2. The average value of *SD*_*measured*_ was 2.0 mm. Although *SD*_*intra*_ was below 1.0 mm in almost all the segments, the values of 1.1–1.7 mm were obtained for some superior and inferior segments.

**Table 2:** Standard deviations of the prostate deformation in the manually defined segments.

	Prostate segment											
	S-A	S-P	S-L	S-R	M-A	M-P	M-L	M-R	I-A	I-P	I-L	I-R
*SD*_*measured*_ (*mm*)	2.7	2.2	2.4	2.6	1.4	1.8	1.6	1.5	2.1	2.0	1.8	2.0
*SD*_*intra*_ (*mm*)	1.3	1.1	1.5	1.7	0.6	0.7	0.8	0.9	0.9	1.0	1.0	1.2
*SD*_*actual*_ (*mm*)	2.4	1.9	1.9	1.9	1.2	1.6	1.3	1.2	1.9	1.7	1.5	1.5

*Abbreviations*: superior-anterior segment: S-A; superior-posterior segment: S-P; superior-right segment: S-R; superior-left segment: S-L; middle-anterior segment: M-A; middle–posterior segment: M-P; middle–right segment: M-R; middle–left segment: M-L; inferior-anterior segment: I-A; inferior–posterior segment: I-P; inferior–right segment: I-R; inferior–left segment: I-L.

Table 3 contains the prostate displacement statistics for all the patients. In the A-P and S-I directions, a prostate displacement of <3 mm was observed in >70% of cases, whereas a prostate displacement of ≥5 mm was observed in <10% of cases. The prostate displacement was below 1.5 mm in the R-L direction.

**Table 3:** Distribution of the displacement values of the prostate CoG according to magnitudes and directions.

	Occurrence frequency, %			
Motion direction	<1 mm	1 mm to 3 mm	3 mm to 5 mm	≥5 mm
R-L	87.4	12.6	0.0	0.0
A-P	30.3	42.9	19.3	7.6
S-I	44.5	35.3	17.6	2.5

*Abbreviations*: right-left direction: R-L; anterior-posterior direction: A-P; superior-inferior direction: S-I.

The multiple regression analysis revealed that the A-P displacement of the CoG of the prostate had a highly significant correlation with the deformations in the M-A (*p* < 0.01) and M-P (*p* < 0.01) segments of the prostate surface (*R* ^*2*^ = 0.84). However, there was no significant correlation between the displacement of the CoG and the deformation of the prostate surface in any other segment.

## Discussion

In this study, we evaluated the deformation of the prostate using a simple method that defined the deformation direction of the prostate and analyzed the correlation between the displacement in the CoG of the prostate and the deformation of its surface in radiation therapy.

The *SD*_*actual*_ of the prostate deformation in all the segments was about 1.5 mm, and the maximum prostate deformation was 13 mm in this study. In this regard, van der Wielen *et al*. implanted three or four fiducial markers in each patient and performed a repeat CT scan before or after a treatment fraction in treatment weeks 2, 4, and 6. As a result, the SD of the obtained prostate deformation was reported to be about 1 mm [10]. In agreement, in the study of Nichol *et al*. who also used fiducial markers and performed MRI before or during a treatment fraction, the SD of the prostate deformation was approximately 1 mm, and the maximum prostate deformation was 13 mm [11]. Although the maximum prostate deformation in the present study was similar to that in the published reports, the corresponding SD was slightly larger. This deviation may result from the differences between our method of analysis and the DIR method employed by van der Wielen *et al*. and Nichol *et al*. A multi-institution deformable registration accuracy study [12] has demonstrated that the mean absolute error and the absolute SD for the prostate ranged from 0.4 to 6.2 mm and from 0.3 to 3.4 mm, respectively. The accuracy of the DIR method varies depending on the algorithm [12], and the point-to-point correspondence between the two images is usually unknown. Moreover, there is no gold standard for the evaluation of the performance of these algorithms [14]. Therefore, at present it may be difficult to analyze organ deformation with sufficient accuracy using the DIR method. In contrast, we utilized a method with a simple deformation model that does not use DIR, and the influences of the prostate volume change and intra-observer error were taken into account. Hence, our methodology did not suffer from potential inaccuracies resulting from differences in the DIR algorithms. We believe that the slightly larger value of the *SD*_*actual*_ of the prostate deformation is a consequence of application of this less biased approach. Actually, the prostate did not deform along a defined direction. It is likely that the final deformation vector is determined by the internal pressure associated with prostate shrinking or swelling and external pressure, for example, from rectum filling, and an algorithm that takes these parameters into account is required for the most reliable evaluation.

**Fig 4:**
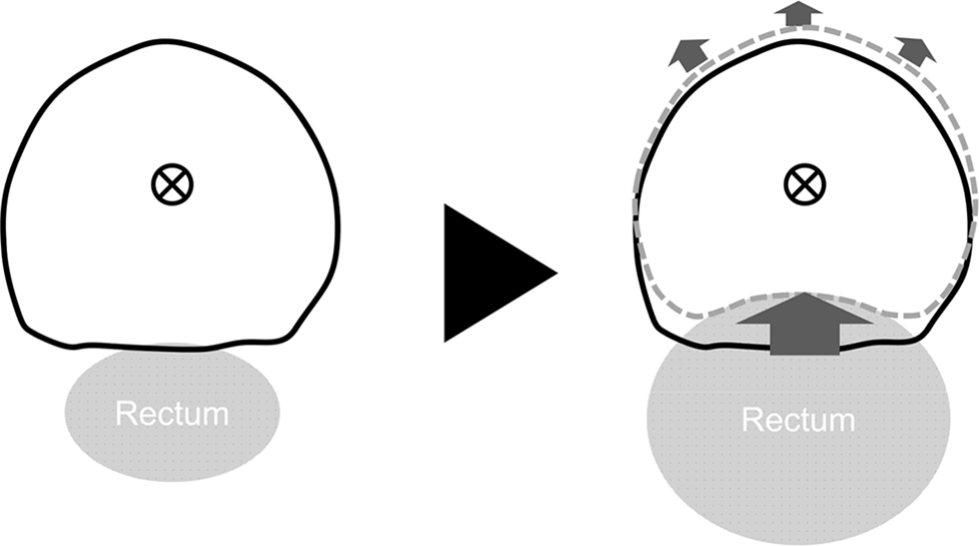
A schematic of prostate deformation obtained by aligning of the planning CT and each CBCT scan based on the center of gravity of the prostate translation. Expansion or displacement of the rectum affects the deformation of the M-P and M-A segments of the prostate.

We found no significant correlation between the displacement of the CoG and the local deformation of the prostate in almost all the segments of the prostate. Therefore, in contrast with the displacement of the CoG, prostate deformations are likely to be mostly random. However, the A-P displacement of the CoG of the prostate had a highly significant correlation with the deformations in the M-A (*p* < 0.01) and M-P (*p* < 0.01) segments. It is known that displacement of the upper rectum significantly affects displacement of the prostate [15], and such expansion or displacement may also influence prostate deformation (Fig 4). Even in the case of IGRT for prostate cancer using implant markers or CBCT, an appropriate IM will have to be added to CTV because of the prostate deformation that is independent of its displacement. Moreover, if the displacement of the prostate occurs in the A-P direction, it may be possible to adjust the IM based on the position of the CoG. For example, if the prostate moved in the anterior direction, a smaller IM (e.g. zero margin) can be used in radiation therapy for prostate cancer. Wen et al. reported that the predicted normal tissue complication probability for late rectal bleeding was reduced by 3.6% on average when the margin was reduced from 10 mm and 6 mm at the prostate/anterior rectal wall interfaces to 5 mm and 3 mm, respectively. Moreover, it further decreased by 1.0% on average when the margin was uniformly reduced to 3 mm [16]. Therefore, even a small margin reduction may substantially reduce the risk of late rectal bleeding, and there is a high probability that radiation therapy with the IM adjusted based on the position of the CoG will be efficient in reducing the margin.

Optimization of the PTV margin that takes into account deformation of the prostate correlated with its displacement will be the topic of our future studies. For example, availability of treatment plan templates with variable PTV margins would allow selecting an appropriate plan for every position of the prostate based on IGRT. Our method may be useful for such adaptive radiotherapy for prostate cancer.

## Conclusion

In this study, we evaluated the deformation of the prostate using a simple method that defined the direction of deformation and evaluated the correlation between the displacement in the CoG of the prostate and the deformation of its surface. Our approach resolves the problem of variable accuracy of DIR algorithms. Prostate deformation takes place even when the average prostate volume remains unchanged during the course of radiation therapy. Our study revealed that A-P displacement of the CoG of the prostate is highly correlated with its deformation in the M-A and M-P segments. IGRT-guided optimization of the treatment plan for every position of the prostate is required for increasing the efficiency of radiation therapy for prostate cancer.
